# Species‐specific responses to white‐nose syndrome in the Great Lakes region

**DOI:** 10.1002/ece3.10267

**Published:** 2023-07-09

**Authors:** Elyse C. Mallinger, Katy R. Goodwin, Alan Kirschbaum, Yunyi Shen, Erin H. Gillam, Erik R. Olson

**Affiliations:** ^1^ Department of Natural Resources Northland College Ashland Wisconsin USA; ^2^ Department of Biological Sciences, Dept. 2715 North Dakota State University Fargo North Dakota USA; ^3^ Great Lakes Inventory and Monitoring Network National Park Service Ashland Wisconsin USA; ^4^ Department of Electrical Engineering and Computer Science, Laboratory for Information and Decision Systems Massachusetts Institute of Technology Cambridge Massachusetts USA

**Keywords:** acoustic activity, bats, disease, latitude, *Pseudogymnoascus destructans*

## Abstract

White‐nose syndrome is a fungal disease that is threatening bat populations across North America. The disease primarily affects cave‐hibernating bats by depleting fat reserves during hibernation and causing a range of other physiological consequences when immune responses are suppressed. Since it was first detected in 2006, the disease has killed millions of bats and is responsible for extensive local extinctions. To better understand the effects of white‐nose syndrome on various bat species, we analyzed summer acoustic survey data collected from 2016 to 2020 at nine US National Parks within the Great Lakes region. We examined the effect that white‐nose syndrome, time of the year relative to pup volancy, habitat type, and regional variation (i.e., park) have on the acoustic abundance (i.e., mean call abundance) of six bat species. As expected, little brown bat (*Myotis lucifugus*) and northern long‐eared bat (*Myotis septentrionalis*), both hibernating species, experienced a significant decline in acoustic abundance following white‐nose syndrome detection. We observed a significant increase in acoustic abundance as white‐nose syndrome progressed for hoary bats (*Lasiurus cinereus*) and silver‐haired bats (*Lasionycteris noctivagans*), both migratory species that are not impacted by the disease. Contrary to our predictions, we observed an increase in big brown bat (*Eptesicus fuscus*; hibernating) acoustic abundance and a decrease in eastern red bat (*Lasiurus borealis*; migratory) acoustic abundance following the detection of white‐nose syndrome. We did not observe any significant changes after the onset of white‐nose syndrome in the seasonal patterns of acoustic activity related to pup volancy, suggesting that production or recruitment of young may not be affected by the disease. Our results suggest that white‐nose syndrome is affecting the acoustic abundance of certain species; however, these changes may not be a result of reduced reproductive success caused by the disease. In addition, species population dynamics may be indirectly affected by white‐nose syndrome as a result of reduced competition or a foraging niche release. We also found that for parks located at higher latitudes, little brown bat and northern long‐eared bat were more likely to experience greater declines in acoustic abundance as a result of white‐nose syndrome. Our work provides insight into species‐specific responses to white‐nose syndrome at a regional scale and examines factors that may facilitate resistance or resiliency to the disease.

## Graphical abstract

To better understand the effects of white‐nose syndrome on various bat species, we performed acoustic surveys from 2016 to 2020 at nine US National Parks within the Great Lakes region. We examined the effect that white‐nose syndrome, time of the year relative to pup volancy, habitat type, and regional variation have on the acoustic abundance of six bat species. Our work represents one of the first comprehensive regional analyses of the species‐specific responses to white‐nose syndrome for national parks.

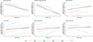

## 1 INTRODUCTION

White‐nose syndrome is a disease caused by the fungus *Pseudogymnoascus destructans*, which infects the skin of hibernating bats and is becoming an increasing threat to bat populations across North America. White‐nose syndrome was first detected in the USA in 2006 near Albany County, New York (Blehert et al., 2009). The disease spread rapidly and has now been detected in 38 US states and eight Canadian provinces (USFWS, 2022b). The infection affects bats primarily during hibernation when immune responses are suppressed (Bouma et al., 2010), resulting in a range of physiological consequences including wing damage (Meteyer et al., 2009; Reichard & Kunz, 2009), excessive dehydration (Cryan et al., 2010), fat store depletions (Blehert et al., 2009), and altered torpor‐arousal cycles (Reeder et al., 2012; Warnecke et al., 2013). White‐nose syndrome has caused extensive local extinctions (Frick et al., 2015) and a decline in some species exceeding 95% (Cheng et al., 2021; Hoyt et al., 2021).

Bats that survive white‐nose syndrome during hibernation may continue to face the repercussions of the disease. Wing damage from white‐nose syndrome has been linked to a lower body mass index, possibly reflecting on an individual's ability to forage (Reichard & Kunz, 2009), and in turn, its ability to survive. In addition, bats that have recovered from white‐nose syndrome may experience reduced reproductive success (Francl et al., 2012; Pettit & O'Keefe, 2017). Adult female bats begin hibernation with greater fat reserves and deplete them more slowly than males by spending more time in torpor (Jonasson & Willis, 2011). However, torpor patterns are altered in individuals infected with white‐nose syndrome, leading to more frequent arousals (Reeder et al., 2012). Therefore, infected female bats may not have enough fat reserves at the end of hibernation to reproduce successfully (Pettit & O'Keefe, 2017). Pettit and O'Keefe (2017) found that white‐nose syndrome is impacting reproduction in little brown bats (*Myotis lucifugus*), northern long‐eared bats (*Myotis septentrionalis*), Indiana bats (*Myotis sodalis*), and big brown bats (*Eptesicus fuscus*). Specifically, after exposure to white‐nose syndrome, they observed an increase in the proportion of captured bats that were nonreproductive, and a decrease in the proportion of captured bats that were lactating, suggesting that female fecundity is negatively affected by the disease (Pettit & O'Keefe, 2017). In another study, captures of juvenile northern long‐eared bats declined significantly after the arrival of white‐nose syndrome (Reynolds et al., 2016). Thus, white‐nose syndrome likely has both direct (direct mortality) and indirect (reduced recruitment) effects for some bat species.

In the western Great Lakes region of the USA, various bat species are experiencing rapid population declines as a result of white‐nose syndrome. In central Indiana, USA, Pettit and O'Keefe (2017) observed an 80% decline in little brown bat, 13% decline in tricolored bat (*Perimyotis subflavus*), and 60% decline in Indiana bat (*Myotis sodalis*) just 4 years after the detection of white‐nose syndrome. Similarly, within 6 years of white‐nose syndrome detection, Kurta and Smith (2020) found that the overall size of the hibernating bat population in mines in northern Michigan had decreased by 90%. In addition, it is predicted that the little brown bat, possibly one of the most abundant species in northern Wisconsin, USA (Jackson, 1961; Long, 2008), could soon experience local extinction in the region (Frick et al., 2010; Huebschman, 2019; Pettit & O'Keefe, 2017).

Understanding the effects of white‐nose syndrome is crucial to the conservation of many bat species. As the disease rapidly continues to spread, it is important for researchers to have effective and efficient monitoring techniques. Acoustic surveys are an effective method for detecting bats and tracking changes in bat activity over time (O'Farrell & Gannon, 1999). Passive acoustic monitoring is advantageous when studying large geographic areas because it is time and cost‐efficient (Coleman et al., 2014), and this method has already proven successful in understanding the impacts of white‐nose syndrome on bat populations (Bombaci et al., 2021; Brooks, 2011).

Our objective was to examine how acoustic abundance of six bat species changed in relation to the onset of white‐nose syndrome. We used acoustic survey data collected over five summers at nine US National Parks spread throughout the western Great Lakes region. We examined how each species of bat responded to the onset of white‐nose syndrome, as well as how location, time of year, and habitat type influenced the response to white‐nose syndrome. We expected acoustic abundance of little brown, big brown, and northern long‐eared bats to have a negative relationship with time since onset of white‐nose syndrome. These three species are hibernating bats that have shown declines in response to white‐nose syndrome elsewhere (Dzal et al., 2011; Ford et al., 2011; Francl et al., 2012; Table 1). In contrast, we expected hoary (*Lasiurus cinereus*), silver‐haired (*Lasionycteris noctivagans*), and eastern red bats (*Lasiurus borealis*) to show either neutral or positive responses to time since onset of white‐nose syndrome because they are migratory bats and less likely to be exposed to white‐nose syndrome during hibernation, and because the disease has not been detected in any of these species (USFWS, 2022a). Migratory bat populations exhibit variable, but generally less substantial responses to the onset of white‐nose syndrome elsewhere (Faure‐Lacroix et al., 2020; Ford et al., 2011; Table 1). However, migratory species are more susceptible to mortality from wind energy development (Kunz et al., 2007), so we may see declines unrelated to white‐nose syndrome. We included the migratory species in our study even though they are less affected by white‐nose syndrome, because their responses can help us rule out other “global” factors that could cause uniform declines in bat populations regardless of species. Including migratory species may also be beneficial in identifying whether or not we detect a disease‐mediated competitive release, as proposed by Bombaci et al. (2021).

**TABLE 1:** A priori model list and hypothesized effect on acoustic abundance of six different bat species of the western Great Lakes region of USA.

Parameter	Little brown bat^a^	Northern long‐eared bat^a^	Big brown bat^a^	Hoary bat^b^	Silver‐haired bat^b^	Eastern red bat^b^
WNS	Decrease^c^			No decrease^d^		
Pup	Increase post‐volancy^e^					
Habitat	No change^f^	Higher in forest^f^ ^,^ ^g^ ^,^ ^h^	Higher in open^i^	Higher in open^j^ ^,^ ^k^	Higher in open^h^, forest/open^l^	Higher in open^i^ ^,^ ^m^ ^,^ ^n^
Park	Variable					
Park*WNS	Decrease, vary by park			No decrease		
Pup*WNS	No increase post‐volancy^e^ ^,^ ^o^ ^,^ ^p^			Increase post‐volancy		
Habitat*WNS	Less decrease in preferred habitat (see above)			Higher in preferred habitat		
Park*Pup	Increase post‐volancy, vary by park					
Habitat*Pup	Lower in open habitat pre‐volancy					

Abbreviations: Habitat, which of the five landcover types were most prevalent in a 200‐m radius of the site: developed, open, forest, open/forest, woody wetland; Park, national park location; Pup, pre‐ or post‐pup volancy; WNS, time since white‐nose syndrome was first detected in an overlapping or adjacent county.

^a^ Hibernating bats.

^b^ Migratory bats.

^c^ Turner et al. (2011).

^d^ Jachowski et al. (2014).

^e^ Ford et al. (2011).

^f^ Van Zyll de Jong (1985).

^g^ Menzel et al. (2002).

^h^ Patriquin and Barclay (2003).

^i^ Loeb and O'keefe (2006).

^j^ Aldridge and Rautenbach (1987).

^k^ Fenton (1990).

^l^ Kunz (1999).

^m^ Ellis et al. (2002).

^n^ Menzel et al. (2005).

^o^ Brooks (2011).

^p^ Dzal et al. (2011).

Due to regional variation in landscape ecology, availability and quality of local hibernacula, and park management, we expected to see variation in bat responses to white‐nose syndrome from park to park. Thus, we expected some bat species may do particularly well in certain parks or that the effects of white‐nose syndrome may vary with park identity. For example, Seltmann et al. (2017) found that chronic stress as a result of habitat disturbance can impact bat immunity; therefore, bats inhabiting parks with greater habitat disturbance may be more susceptible to mortality from white‐nose syndrome. In addition, the rate at which white‐nose syndrome spreads may be higher in parks with a larger number of hibernacula or parks that experience longer winters (Maher et al., 2012; Table 1). Habitat type is an important predictor of occupancy for different bat species (Pauli et al., 2017); thus, we also expected acoustic abundance to vary within each park at the habitat level. We predicted that species would be recorded more often in their preferred habitat types and would potentially be more resilient to white‐nose syndrome at sites containing those preferred habitats (Table 1). We also predicted an interaction between habitat type and time of year. For example, prior to pups becoming volant, female bats may forage closer to the roost site in order to care for young during lactation (Henry et al., 2002). Because roosts are typically located in forests (Drake et al., 2020), we predicted that, in open habitat, we would detect less bat activity before pup volancy than after pup volancy (Table 1). However, some bat species are primarily open space foragers (Fenton, 1990), so this seasonal effect may not hold true for all species.

At the seasonal level, we expected an increase in acoustic activity from early summer to late summer for all species prior to white‐nose syndrome, reflecting the addition of juveniles to the population (Ford et al., 2011). Following the onset of white‐nose syndrome, for hibernating species, we expected no significant increase in acoustic activity from early summer to late summer, reflecting reduced reproductive success related to white‐nose syndrome. For migratory species, we expected late summer increases in acoustic activity regardless of the onset of white‐nose syndrome (Brooks, 2011; Dzal et al., 2011; Ford et al., 2011; Table 1).

We used generalized linear mixed models and model ranking within an information theoretic framework (Burnham & Anderson, 2002) to test our hypotheses (Table 1) and identify important parameters shaping bat acoustic abundance within the region.

## 2 METHODS

### 2.1 Study area

**FIGURE 1:**
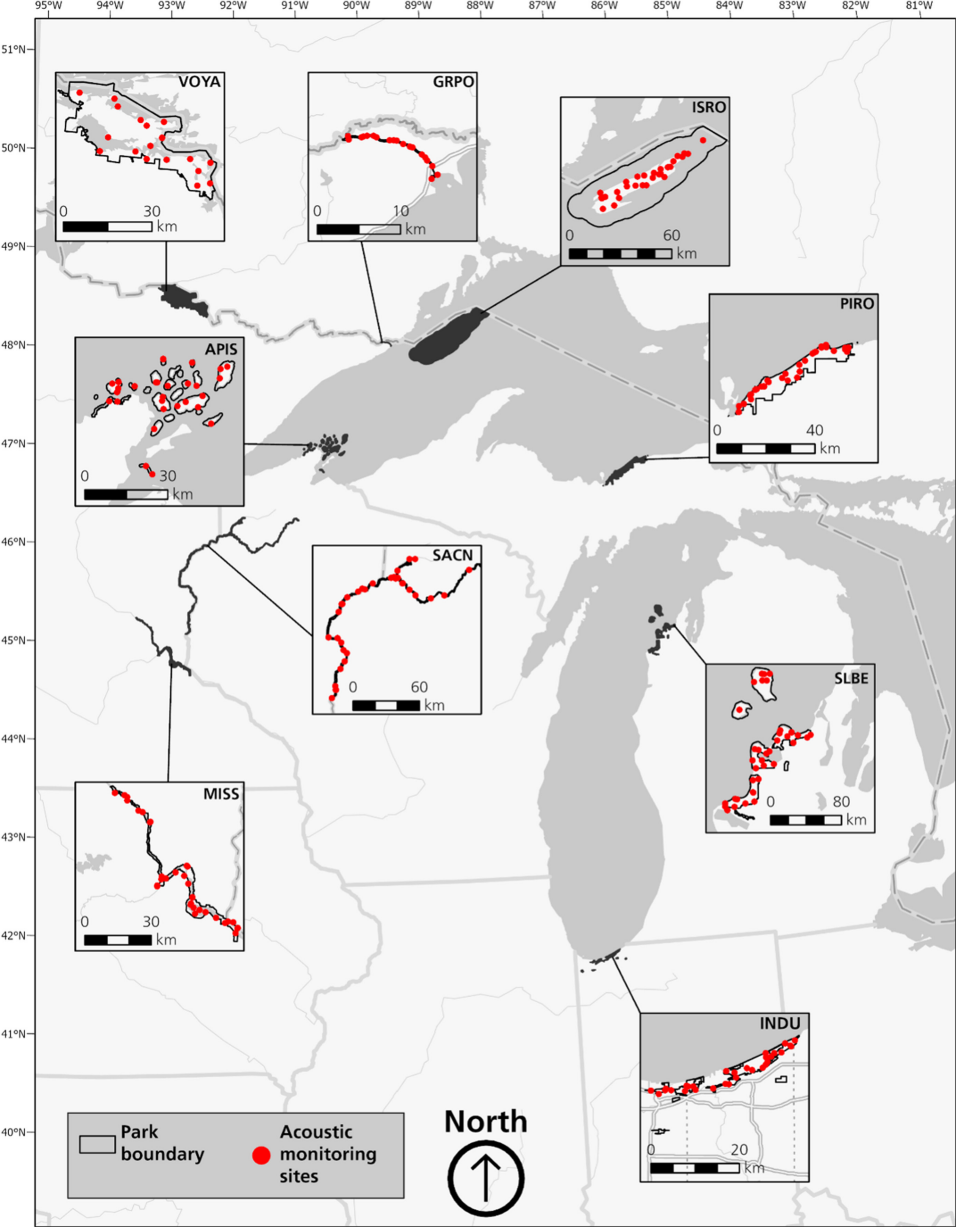
Distribution of bat acoustic monitoring sites in nine national parks of the western Great Lakes region, USA (2016–2020).

We conducted acoustic monitoring at nine US National Parks throughout the western Great Lakes region: Apostle Islands National Lakeshore (APIS), Grand Portage National Monument (GRPO), Indiana Dunes National Park (INDU), Isle Royale National Park (ISRO), Mississippi National River and Recreation Area (MISS), Pictured Rocks National Lakeshore (PIRO), Saint Croix National Scenic Riverway (SACN), Sleeping Bear Dunes National Lakeshore (SLBE), and Voyageurs National Park (VOYA; Figure 1). These parks contain diverse habitat types including boreal forest, floodplain forest, wetlands, beaches and sand dunes, oak savanna, and grasslands. Parks also vary in size, shape, and regional location (Figure 1).

Nine species of bats have ranges overlapping our study area (Kurta, 2017), including five hibernating species that are susceptible to white‐nose syndrome: little brown bat, big brown bat, northern long‐eared bat, Indiana bat, and tricolored bat. The other four species, eastern red bat, hoary bat, silver‐haired bat, and evening bat (*Nycticeius humeralis*) are migratory species and have not shown symptoms of the disease (USFWS, 2022a). We limited our analyses to the six species of bats found at all nine National Parks in our study area; the remaining species have more limited ranges and were detected infrequently. Our six species included three hibernating species (little brown bat, big brown bat, northern long‐eared bat) and three migrating species (hoary bat, eastern red bat, silver‐haired bat).

### 2.2 Field methods

We divided each of the nine national parks into a grid of 1‐km^2^ cells and selected cells for sampling using the Generalized Random Tessellation Stratification method to obtain a sample that was both randomized and spatially balanced (Larsen et al., 2008; Stevens & Olsen, 2004). Within each cell, we chose a specific monitoring site based on accessibility and suitable bat habitat. We defined suitable habitat as areas where bats are likely to travel or forage, such as trail corridors, forest edges, and wetlands. We conducted sampling at 18–35 sites per park, with sites revisited yearly. In a few cases, safety issues or lack of access prevented us from returning to the same site, so we moved the site to a different location within the cell. Full details of sample design and site selection are available in Goodwin et al. (2020).

We used Song Meter SM3BAT and SM4BAT‐FS (Wildlife Acoustics) and Pettersson D500X (Pettersson Elektronik AB) detectors to passively record bat echolocation passes in full spectrum. The detector brand used at each park was consistent throughout the study and only one park used Pettersson detectors (PIRO). We mounted ultrasonic microphones directly to the detector or on a separate tripod or pole, at least 1 m above the ground. We programmed detectors to record every night from 18:00 to 08:00, except one park (PIRO) which recorded from 30 min before sunset to 15 min after sunrise. We deployed one detector at each site for a period of 7–14 nights at some point between June 1st and August 15th for each year of sampling. All nine parks were sampled in 2016–2018, seven parks were sampled in 2019, and five parks were sampled in 2020.

We processed acoustic recordings using Kaleidoscope Pro software (Wildlife Acoustics) to filter out noise and assign species classifications. We used the classifier Bats of North America 3.1.0 with the sensitivity level set to −1 More Sensitive/Liberal. We processed data for each park separately in order to customize the list of potential species used by Kaleidoscope Pro during classification. We allowed Kaleidoscope Pro to consider only the 6 to 9 species with known ranges within each park's boundaries based on published range maps (Harvey et al., 2011; Kurta, 2017; Rodhouse et al., 2016). Previous studies indicate that Kaleidoscope Pro results may include some misclassifications (Brabant et al., 2018; Goodwin & Gillam, 2021). This issue is often addressed by manually reviewing call files to verify species classifications; however, given the magnitude of our dataset this was not feasible. Recordings that occurred outside of protocol dates and deployments with equipment failure were excluded from the analysis.

### 2.3 Data analysis

We examined the effects of four independent variables on bat acoustic activity: years since white‐nose syndrome was first detected, time of year in relation to pup volancy, habitat type, and park. We fit generalized linear mixed models and followed a model ranking and information theoretic approach (Burnham & Anderson, 2002).

Our dependent variable was bat acoustic abundance, defined as the mean number of call files per recording night. Researchers have demonstrated that acoustic data can provide an estimate of actual density for bats (Revilla‐Martín et al., 2021; Whiting et al., 2022). We defined a “call file” as any audio file containing at least one bat echolocation sequence and that is identifiable to the species level by the software Kaleidoscope Pro. We used file‐level classification results from Kaleidoscope Pro to calculate the total number of call files identified to a species and divided this by the total number of recording nights. Although we cannot measure absolute abundance or density of bats from acoustic data, we can assess relative changes in bat acoustic activity. We calculated a bat acoustic abundance value for each species at each site in each year of sampling. If a site was sampled both before and after pups became volant, we calculated acoustic abundance for each period separately. We log transformed the acoustic abundance values to meet assumptions of normality and checked for a normal distribution by creating a histogram.

To create the years since white‐nose syndrome was detected variable, we identified the counties overlapping or adjacent to each park that had white‐nose syndrome detection data available (USFWS, 2022b). We selected the county with the earliest detection of white‐nose syndrome and calculated the difference between each year of our acoustic survey and the year of first detection. This calculation produced a set of numbers ranging from zero through eight, with zero meaning white‐nose syndrome was first detected in the same year as the survey, and eight meaning white‐nose syndrome had been present for at least 8 years.

To evaluate the relationship between bat acoustic abundance and the onset of volancy in juvenile bats, we divided the summer season into two different time periods: pre‐ and post‐volancy. We considered echolocation call files recorded prior to July 15 to have occurred prior to the onset of volancy in juveniles (pre‐volant period) and files recorded on or after July 15 to have occurred after the onset of volancy (post‐volant period). We based the pre‐ and post‐volant periods on the estimated timing of pup birth and development which is similar across all six species we analyzed (Adams, 1997; Koehler & Barclay, 2000; Kunz, 1971; McClure, 1942; O'Shea et al., 2021).

For our habitat variables, we calculated the percentage of landcover classes within a 200‐m buffer around each survey site using the National Land Cover Database for 2016 (Dewitz, 2019). We then identified the dominant landcover classes based on percentage of area within 200 m (i.e., what landcover types had the greatest percent cover, >35%, within 200 m) for each site and then we identified the most common dominant landcover types, which included: developed (i.e., developed low, medium, and high landcover types), open (i.e., developed open, open water, barren land, cultivated crops, hay/pasture, herbaceous, scrub/shrub landcover types), woody wetland (provides unique foraging habitat for bats and was consistently dominant, thus, includes only woody wetland landcover types), forest (i.e., deciduous, evergreen, and mixed forest landcover types), and open forest (i.e., sites that had both open and forest or woody wetland landcover types as the most dominant landcover types at >25% for each and a combined total of >70%; e.g., forest edges or sparsely forested areas). In addition, to account for regional variation in bat acoustic activity relative to ecosystem type, we included park as an independent variable.

For each species, we constructed 29 a priori models, namely four single variable models, 23 interactive or additive biologically relevant models, a null model, and a global model (Table S1). For all models, we included site as a random effect to account for site‐level variation. We ranked model importance according to second‐order Akaike information criterion (AIC_c_; Adelman et al., 2015; Allen et al., 2019). Using the conditional probabilities (AIC_c_ weight), we determined the optimal model(s) (AIC_c_ weight > 0; Olson et al., 2019). We generated full model‐averaged coefficients (Barton, 2022) across all models to identify the effect of the predictor variable. For each variable, we reported the beta coefficient along with the 95% confidence intervals. A variable was considered to be a significant predictor if the corresponding 95% confidence intervals did not span zero (Olson et al., 2019). To assess goodness of fit of the final models we examined the histogram of the residuals and the QQ plot. We also calculated the conditional and marginal coefficients of determination computed with the function R‐squared GLMM (Barton, 2022). The marginal R‐squared represents the variance explained by the fixed effects, while the conditional R‐squared represents the variance explained by both the fixed and random effects (Nakagawa & Schielzeth, 2013). We also assessed the magnitude of the site‐level random effect by calculating standard deviation. Analyses were done using R package *MuMIn* (Barton, 2022) in R 3.6.1 (R Core Team, 2019).

We calculated the percent change in number of call files for all species at all parks. Although all parks were surveyed in 2016, some were not surveyed in 2019 or 2020; thus, we used the total number of call files from 2016 and the last year surveyed to determine the percent change within a park. We also averaged the number of call files across parks and determined the percent change in call files for each species from 2016 to 2020. Parks that were not surveyed in 2020 were excluded from this part of the analysis. In addition, we calculated the percent change in mean number of call files per detector night from the pre‐volancy period to the post‐volancy period for each species. Finally, after observing the results of the prior steps, we performed an *a posteri* linear regression to examine the relationship between the latitude of a park and the slope of the interaction between white‐nose syndrome and park from the full model‐averaged coefficients results for the two hibernating species that exhibited the most significant negative response to years since white‐nose syndrome detection, that is, little brown bat and northern long‐eared bat.

## 3 RESULTS

From 2016 to 2020, a total of 1,512,168 bat call files were recorded across all parks. Big brown bat calls accounted for 24% of total call files, followed by little brown bat (21%), silver‐haired bat (20%), eastern red bat (16%), hoary bat (14%), and northern long‐eared bat (1%), respectively. The remaining call files were either unidentified or from evening bat, tricolored bat, or Indiana bat, species that were excluded from our analysis due to low sample size. Indiana Dunes National Park had the most call files (417,930), whereas Pictured Rocks National Lakeshore had the least (23,847). Overall, 55% of call files were recorded during the pre‐volancy period (before July 15) and 45% of call files were recorded during the post‐volancy period (on or after July 15).

Averaged across parks, the mean number of call files per night for little brown bat declined by 83% from 2016 to 2020 (Table 2). Averaged across parks and years, the mean number of little brown bat call files per night increased by 39% from pre‐ to post‐volancy. The top model predicting little brown bat acoustic abundance included pup volancy period and the interaction between park and years since white‐nose syndrome (AIC_c_ weight = 0.82; Table 3). Model‐averaged coefficients indicated that little brown bat acoustic abundance had a significant negative correlation with number of years since white‐nose syndrome was detected (β = −0.318 ± 0.169; Table S2). However, there was regional variation in acoustic abundance as some parks had significant slope and intercept adjustments (Table S2). Little brown bat acoustic abundance was significantly lower during the pre‐volancy period than the post‐volancy period (β = −0.072 ± 0.052) but this relationship did not change with time since white‐nose syndrome (Table S2).

**TABLE 2:** Percent change since 2016 in mean number of recorded calls per detector night of six different bat species.

Park	Little brown bat	Northern long‐eared bat	Big brown bat	Hoary bat	Silver‐haired bat	Eastern red bat
VOYA (2020)^a^	−75.2%	−83.7%	119.2%	1930.4%	301.0%	−42.1%
ISRO (2020)^a^	−94.4%	−95.6%	25.0%	17.7%	418.2%	−59.1%
GRPO (2020)^a^	−91.4%	−99.1%	87.0%	139.0%	471.3%	−78.1%
APIS (2019)^a^	−48.4%	−55.4%	20.6%	−69.6%	142.9%	−34.6%
PIRO (2018)^a^	−87.0%	−96.2%	−56.0%	55.6%	−54.5%	−86.9%
SACN (2020)^a^	−75.1%	−93.1%	64.3%	90.1%	109.4%	236.7%
SLBE (2019)^a^	−12.3%	−8.1%	−1.4%	−11.5%	−24.0%	−74.3%
MISS (2019)^a^	−40.4%	−76.7%	−9.0%	−3.2%	2.5%	−12.9%
INDU (2020)^a^	−46.1%	−11.4%	69.5%	34.0%	98.6%	−41.2%
All parks^b^	−82.8%	−94.4%	72.8%	201.8%	158.6%	−0.9%

*Note*: Data were collected at nine national parks in the western Great Lakes region of USA between 2016 and 2020. Parks are listed in order of latitude, from north to south.

Abbreviations: APIS, Apostle Islands National Lakeshore; GRPO, Grand Portage National Monument; INDU, Indiana Dunes National Park; ISRO, Isle Royale National Park; MISS, Mississippi National River and Recreation Area; PIRO, Pictured Rocks National Lakeshore; SACN, Saint Croix National Scenic Riverway; SLBE, Sleeping Bear Dunes National Lakeshore; VOYA, Voyageurs National Park.

^a^ Percent change between the mean number of calls recorded in 2016 and the last year surveyed (where last year surveyed is denoted in parentheses).

^b^ Percent change using the mean number of calls recorded in 2016 and 2020. Parks without surveys in 2020 were excluded from this calculation.

**TABLE 3:** Model ranking results for factors influencing acoustic abundance of six bat species from nine national parks within the western Great Lakes region of USA from 2016 to 2020.

Species	Model	*k*	∆AIC_c_	⍵	*R* ^2^m	*R* ^2^c	SD
Little brown bat	Pup + WNS*Park	21	0.00	0.82	0.40	0.76	1.39
	Habitat + Pup + Park*WNS	25	3.59	0.14	0.41	0.76	1.38
	Park*WNS	20	6.34	0.03	0.40	.076	1.40
Northern long‐eared bat	Habitat + Park*WNS	24	0.00	0.53	0.36	0.61	1.06
	Park*WNS	20	1.02	0.32	0.35	0.61	1.08
	Habitat + Pup + Park*WNS	25	3.52	0.09	0.36	0.61	1.06
	Pup + WNS*Park	21	4.55	0.05	0.35	0.61	1.08
Big brown bat	WNS + Park*Pup	21	0.00	0.73	0.65	0.86	1.22
	Habitat + WNS + Park*Pup	25	3.48	0.13	0.66	0.86	1.21
	Pup + WNS + Park	13	4.00	0.10	0.65	0.86	1.22
	Habitat + Pup + WNS + Park	17	6.62	0.03	0.65	0.86	1.21
	Park + WNS*Pup	14	9.20	0.01	0.65	0.86	1.22
Hoary bat	Habitat + WNS + Park	16	0.00	0.66	0.36	0.74	1.34
	Park + WNS	12	2.09	0.23	0.34	0.74	1.36
	Habitat + Pup + WNS + Park	17	4.78	0.06	0.36	0.74	1.34
	Pup + WNS + Park	13	6.76	0.02	0.34	0.74	1.36
	Habitat + Pup*WNS + Park	18	7.74	0.01	0.36	0.74	1.34
	Habitat + WNS + Park*Pup	25	9.35	0.01	0.37	0.74	1.32
Silver‐haired bat	Park*WNS	20	0.00	0.55	0.38	0.78	1.25
	Habitat + Park*WNS	24	0.77	0.37	0.39	0.78	1.24
	Pup + WNS*Park	21	4.82	0.05	0.38	0.78	1.26
	Habitat + Pup + Park*WNS	25	5.52	0.03	0.39	0.78	1.24
Eastern red bat	WNS + Park*Pup	21	0.00	0.77	0.52	0.79	1.37
	Habitat + WNS + Park*Pup	25	2.43	0.23	0.53	0.79	1.36

*Note*: The dependent variable for all models was acoustic abundance, defined as the log mean number of bat call files per detector night. Only models with an AIC_c_ weight greater than zero are included.

Abbreviations: ∆AIC_c_, the difference between model second‐order Akaike's information criterion (AIC_c_) and lowest AIC_c_ in the model set; ⍵, Akaike model weight; Habitat, which of the five landcover types were most prevalent in a 200‐m radius of the site: open, forest, open/forest, woody wetland, developed; *k*, number of estimable parameters; Pup, pre‐ or post‐pup volancy; *R* ^2^c, conditional coefficient of determination; *R* ^2^m, marginal coefficient of determination; SD, standard deviation of the random effect (site); WNS, time since white‐nose syndrome was first detected in an overlapping or adjacent county.

From 2016 to 2020, averaged across parks, the mean number of calls per night for northern long‐eared bat declined by 94% (Table 2). Averaged across parks and years, the mean number of call files per night decreased by 7% from pre‐ to post‐volancy. The top model predicting northern long‐eared bat acoustic abundance included habitat type and the interaction between park and years since white‐nose syndrome (AIC_c_ weight = 0.53; Table 3). Model‐averaged coefficients indicated that northern long‐eared bat acoustic abundance had a significant negative correlation with number of years since white‐nose syndrome was detected (β = −0.371 ± 0.212; Table S3). There was significant regional variation in acoustic abundance as some parks had significant slope and intercept adjustments (Table S3).

Averaged across parks, the mean number of call files per night for big brown bat increased by 73% from 2016 to 2020 (Table 2). Averaged across parks and years, the mean number of call files per night increased by 8% from pre‐ to post‐volancy. The top model predicting big brown bat acoustic abundance included years since white‐nose syndrome and the interaction between park and pup volancy period (AIC_c_ weight = 0.73; Table 3). However, the coefficients for the park and pup volancy period interactions were not significant for any parks. Model‐averaged coefficients indicated that big brown bat acoustic abundance was positively correlated with years since white‐nose syndrome (β = 0.102 ± 0.039; Table S4). Big brown bat acoustic abundance was significantly lower in the pre‐volancy versus post‐volancy period (β = −0.099 ± 0.099) but this relationship did not change with time since white‐nose syndrome (Table S4).

The mean number of hoary bat call files per night increased by 202% from 2016 to 2020, when averaged across parks (Table 2). Averaged across parks and years, the mean number of hoary bat call files per night decreased by 44% from pre‐ to post‐volancy. The top model predicting hoary bat acoustic abundance included habitat type, years since white‐nose syndrome, and park (AIC_c_ weight = 0.66; Table 3). Model‐averaged coefficients indicated that hoary bat acoustic abundance had a significant positive correlation with years since white‐nose syndrome (β = 0.167 ± 0.054; Table S5). However, there was significant regional variation in hoary bat acoustic abundance as the intercept adjustment for most parks was significant (Table S5).

Averaged across parks, from 2016 to 2020 the mean number of silver‐haired bat call files per night increased by 159% (Table 2). Averaged across parks and years, the mean number of call files per night decreased by 16% from pre‐ to post‐volancy. The top model predicting silver‐haired bat activity included habitat type and the interaction between park and years since white‐nose syndrome (AIC_c_ weight = 0.55; Table 3). Model‐averaged coefficients indicated that silver‐haired bat acoustic abundance had a significant positive correlation with white‐nose syndrome years (β = 0.335 ± 0.168; Table S6). However, there was significant regional variation in this relationship as some parks had significant slope and intercept adjustments (Table S6).

The mean number of eastern red bat call files per night, averaged across parks, decreased by 1% from 2016 to 2020 (Table 2). Averaged across parks and years, the mean number of call files per night increased by 159% pre‐ to post‐volancy. The top model for eastern red bat acoustic abundance included white‐nose syndrome years, and the interaction between park and pup volancy period, (AIC_c_ weight = 0.77; Table 3). Model‐averaged coefficients indicated that eastern red bat acoustic abundance had a negative relationship with years since white‐nose syndrome was detected (β = −0.176 ± 0.048; Table S7). Overall, eastern red bat acoustic abundance was significantly lower in the pre‐volancy period (β = −0.199 ± 0.120). However, there was significant variation among parks in the strength of this relationship (Table S7).

**FIGURE 2:**
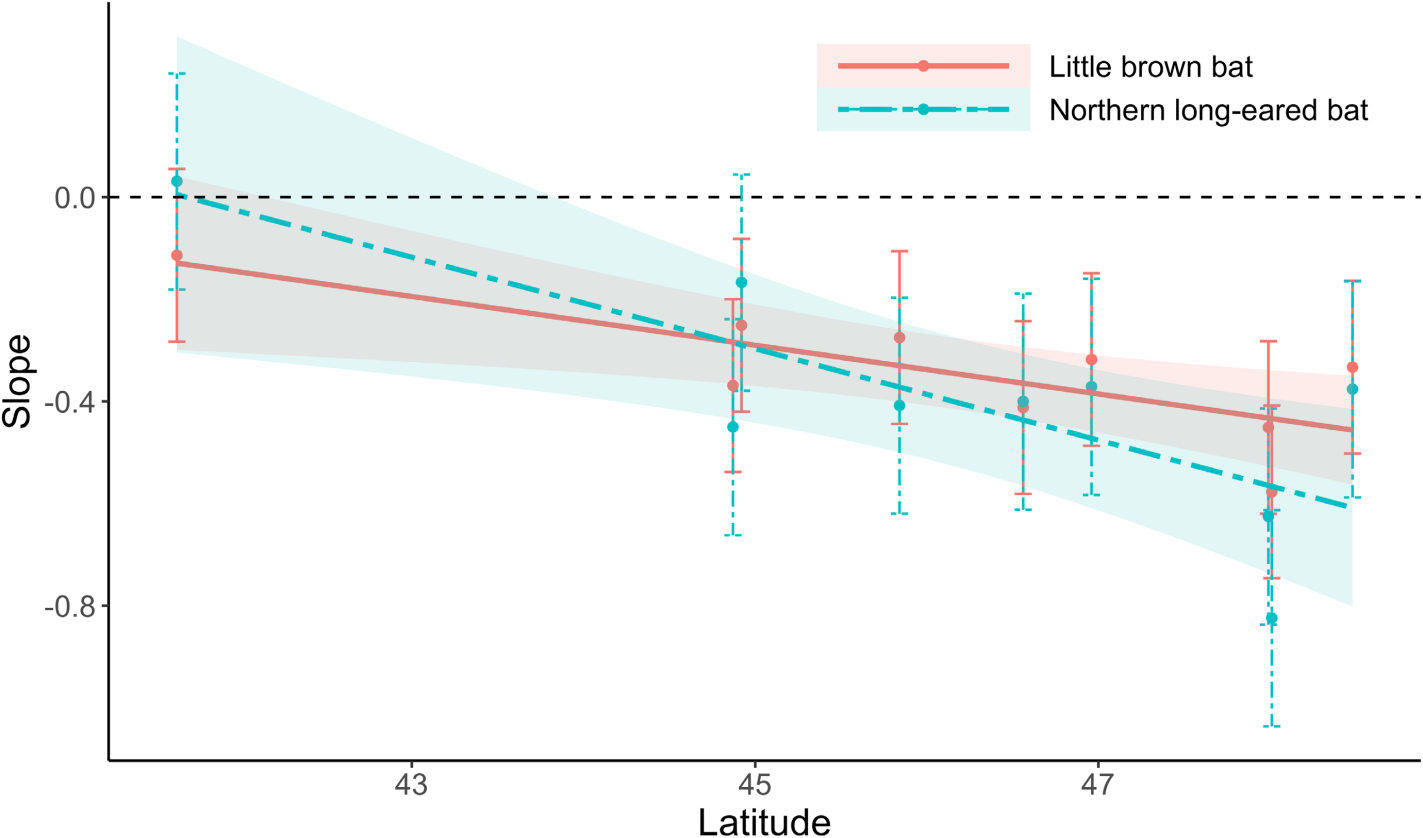
Effect‐size of white‐nose syndrome by park for two of the most negatively affected species, little brown bats (*Myotis lucifugus*) and northern long‐eared bats (*Myotis septentrionalis*), relative to the latitude of each park. Error bars indicate 95% confidence intervals. Acoustic data were collected from nine national parks throughout the western Great Lakes region of the USA from 2016 to 2020.

Regarding timing associated with pup volancy, model‐averaged coefficients indicated that little brown, big brown, and eastern red bat acoustic abundance was significantly higher during the post‐volancy period. However, the interaction between pup volancy period and white‐nose syndrome was nonsignificant for all species (Figure S1, Tables S2–S7), suggesting years since white‐nose syndrome has a limited effect on juvenile recruitment. On the contrary, the interaction between park identity and years since white‐nose syndrome was detected was present and significant in both of our top models for little brown and northern long eared bats—the two species exhibiting the most negative effects associated with years since white‐nose syndrome. After examining the park‐specific slopes and puzzling over what might be the driver of the park‐level variation observed, we hypothesized that there may have been a latitudinal effect. Thus, we implemented an *a posteri* linear regression analysis of the park‐level slopes for years since white‐nose syndrome and park latitude, which resulted in a significant, negative correlation (*p* < .05; Figure 2).

## 4 DISCUSSION

**FIGURE 3:**
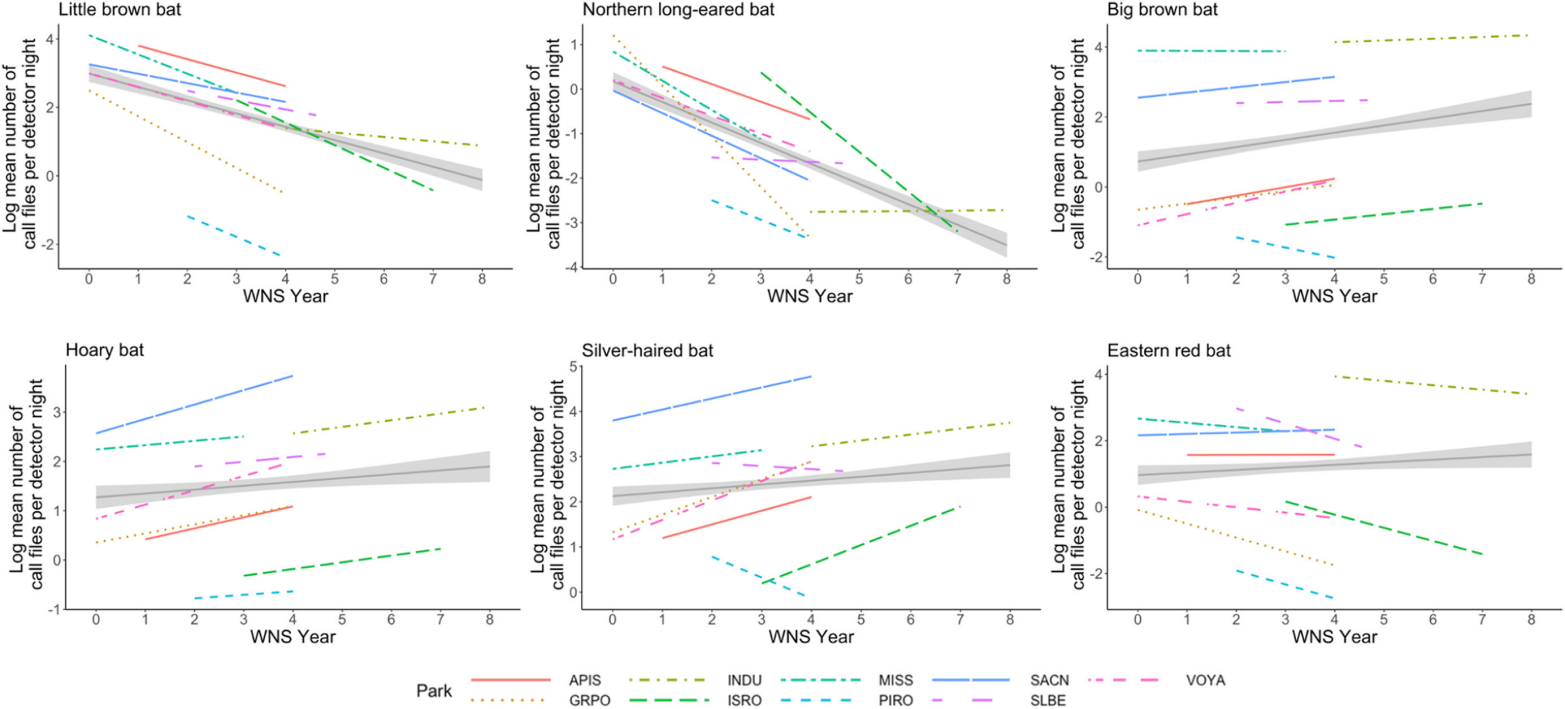
Log mean number of bat calls per detector night relative to years since white‐nose syndrome was first detected (WNS Year) for six different bat species overall (black solid line; gray shaded region = 95% CI) and for each of nine national parks (see legend) throughout the western Great Lakes region, USA (2016–2020). GRPO, Grand Portage National Monument; INDU, Indiana Dunes National Park; ISRO, Isle Royale National Park; MISS, Mississippi National River and Recreation Area; PIRO, Pictured Rocks National Lakeshore; SACN, Saint Croix National Scenic Riverway; SLBE, Sleeping Bear Dunes National Lakeshore; VOYA, Voyageurs National Park. *y*‐axis varies by species.

Our results demonstrate bat species‐specific responses to the progression of white‐nose syndrome across the Western Great Lakes region of USA. We examined the effects that white‐nose syndrome, time of the year relative to pup volancy, park location, and habitat type have on bat acoustic abundance. All six bat species included in our analysis exhibited significant changes in acoustic abundance following the detection of white‐nose syndrome (Figure 3). Two of the hibernating species, little brown bat and northern long‐eared bat, experienced significant declines in acoustic abundance since the onset of white‐nose syndrome. These results are consistent with our predictions and similar to that of other studies. For example, in New York, Dzal et al. (2011) detected a 78% decline in the summer activity levels of little brown bat following the arrival of white‐nose syndrome. In addition, Reynolds et al. (2016) observed a 95% decrease in capture rates for northern long‐eared bats following the detection of white‐nose syndrome in Virginia. Similar trends are seen within the western Great Lakes region of the USA. In central Illinois, Langwig et al. (2015) found that northern long‐eared bats declined by 95%–99% and little brown bats 81%–88% within 2 years. Another study found that the detection probability of northern long‐eared bats declined in both central Wisconsin and the Upper Peninsula of Michigan from 2015 to 2016 (Hyzy et al., 2020), possibly a result of white‐nose syndrome.

By contrast, the big brown bat, although also a hibernating species, increased in acoustic abundance in our study. The number of big brown bat calls increased from 2016 to 2020 and our model results also indicated a significant positive relationship with years since white‐nose syndrome onset. Our findings suggest big brown bats may be less impacted than other hibernating bat species in our study system, and other studies have found similar results. For example, Pettit and O'Keefe (2017) observed a 11.5% increase in big brown bat captures following the year white‐nose syndrome was first detected in Indiana. In central Massachusetts, a 47.8% increase in the number of call sequences was observed for large‐bodied species, with big brown bats comprising 93% of those calls (Brooks, 2011; Morningstar et al., 2019). Likewise, Morningstar et al. (2019) found mean acoustic activity of big brown bats in southern Ontario increased by 27.5% after the arrival of white‐nose syndrome. One possible explanation is that big brown bats have higher body fat content prior to hibernation and therefore are better able to survive resource depletion from white‐nose syndrome (Frank et al., 2014). In addition, big brown bats often hibernate alone or in small groups which may inhibit the spread of white‐nose syndrome (Whitaker & Gummer, 1992). These two hypotheses explain why big brown bats may not be decreasing as a result of white‐nose syndrome, yet they do not speak to the apparent increase in big brown acoustic abundance during the same time frame that other hibernating species are experiencing declines. We suspect this could be explained, in part, by reduced competition given there are fewer little brown and northern long‐eared bats on the landscape post‐white‐nose syndrome. In support of this theory, Morningstar et al. (2019) found that big brown bats consumed a wider variety of prey and many of the insects once consumed by little brown bats following the detection of white‐nose syndrome. Similar to our study, Bombaci et al. (2021) documented a slight increase in big brown bat activity in West Virginia post‐white‐nose syndrome, possibly a result of a competitive release.

Similar to big brown bats, silver‐haired and hoary bats are also experiencing a significant increase in activity following the detection of white‐nose syndrome. Our results are similar to other studies, such as Nocera et al. (2019) who found a 73% increase in hoary bat activity and a 12% increase in silver‐haired bat activity after white‐nose syndrome arrival. Faure‐Lacroix et al. (2020) analyzed silver‐haired and big brown bats as one group due to their similar echolocation calls and found that the number of detections for these species more than doubled since the onset of white‐nose syndrome in Quebec, Canada. Similar to the big brown bat, both hoary and silver‐haired bats may be experiencing an increase in acoustic abundance as a result of reduced competition or foraging niche release. For example, following the detection of white‐nose syndrome in southeastern Ontario, Canada, hoary and silver‐haired bats were more frequently observed in clutter or edge habitats compared to before white‐nose syndrome, possibly a result of relaxed competition allowing them to use more habitat types (Mayberry et al., 2020).

Counter to our expectations, we observed a decline in eastern red bat after the onset of white‐nose syndrome. As a migratory species, we expected this species to have a stable or increasing trend relative to the disease. Our models indicated a significant negative relationship between this species' acoustic abundance and time since white‐nose syndrome. The mean number of calls also decreased at eight out of nine park locations (the exception was Saint Croix National Scenic Riverway which experienced a large increase in number of call files during that time frame). It is possible that our observed decline in eastern red bats was not caused by white‐nose syndrome. For example, in lower Michigan, USA, Winhold et al. (2008) observed a 52%–85% decline in capture rates of the eastern red bat within 12–26 years prior to the onset of white‐nose syndrome. The observed decline could be a result of habitat fragmentation and reduction (Carter et al., 2003), environmental pollutants (Clark Jr & Shore, 2001), or increased mortality from wind energy generation (Arnett et al., 2008; Kunz et al., 2007), however, the scope of our analysis limits our ability to draw any inferences here.

Of the six bat species included in this study, big brown, little brown, and eastern red bats experienced a significant increase in acoustic abundance later in the summer after pups become volant, based on model‐averaged coefficients and the percent change in number of call files. Our findings are indicative of positive juvenile recruitment for those species. However, the interaction between white‐nose syndrome and pup volancy was not significant for any of the species in our study (Figure S1, Tables S1–S6). The observed lack of relationship between white‐nose syndrome progression and recruitment contrasts with other studies. In Virginia, USA, captures of juvenile northern long‐eared bats declined by 77% following the onset of white‐nose syndrome (Reynolds et al., 2016). Furthermore, Ford et al. (2011) found that the activity levels of little brown bat decreased from early to late summer post‐white‐nose syndrome whereas the opposite occurred pre‐white‐nose syndrome, suggesting that fewer volant young are being added to the population. However, our results suggest that the progression of white‐nose syndrome may not be affecting pup production for any of the species and therefore, the decline observed in acoustic activity for species affected by white‐nose syndrome may be primarily a result of disease‐related mortality, not reduced recruitment. However, our study may not be well designed to detect a recruitment effect, and more local studies tracking changes in juvenile recruitment are needed.

Habitat type was included in the top model for predicting the acoustic abundance of two bat species (northern long‐eared bat and hoary bat); however, model‐averaged coefficients indicated that there was not a significant correlation between habitat type and acoustic abundance for any of the species, suggesting that habitat is not as important as other factors. In addition, we did not detect an interaction between habitat type and time since white‐nose syndrome, suggesting that summer habitat type may not be effective at facilitating resistance or resiliency of bat populations to white‐nose syndrome. However, we did detect significant interactions between park and time since white‐nose syndrome, likely a result of the differences in latitude. We detected a significant negative correlation between the slope of the effect of white‐nose syndrome and park latitude for these two species. This correlation suggests that the impact of white‐nose syndrome may be greater at higher latitudes. Vanderwolf and McAlpine (2021), found that fungal growth of white‐nose syndrome is not slowed down by colder hibernacula temperatures, even at its northern range limit. Arousals from torpor by bats in cold hibernacula requires costly thermogenesis, depleting their energy budget (Thomas et al., 1990). It is possible that the colder climates and longer winters associated with overwintering at higher latitudes is causing bats to use up more energy during the more frequent arousals associated with white‐nose syndrome infection (Reeder et al., 2012). Longer hibernation seasons are also associated with higher latitudes (Kunz & Fenton, 2005); therefore, it is possible that little brown bats and northern long‐eared bats at higher latitudes are experiencing greater effects from white‐nose syndrome because they spend more time in hibernation without enough fat stores to survive the entire winter. Interestingly, Maher et al. (2012) found that the rate of spread for white‐nose syndrome was associated with longer winters (Maher et al., 2012), further indication of the importance of winter length in regards to the dynamic interactions between bat life histories and white‐nose syndrome.

Contrary to our predictions, we did not observe an interactive effect between volancy period and habitat type for any of the species. Prior to pup volancy, bats are likely caring for pups and foraging near roosts (Henry et al., 2002) which are typically located in forest habitat (Drake et al., 2020); therefore, we hypothesized that bat acoustic abundance would be lower in open habitats prior to pup volancy. However, lactation is energetically expensive (Anthony & Kunz, 1977); therefore, it is possible that female bats are forced to forage further away from the roosts to find areas with adequate amounts of prey (Menzel et al., 2001). Depending on the availability of resources at a particular location, either of these predictions could be occurring, but overall, we did not detect a significant difference in habitat use pre‐volancy versus post‐volancy.

Our work represents one of the first comprehensive regional analyses of the species‐specific responses to white‐nose syndrome for national parks. National parks are ideal for acoustic monitoring because they are protected habitats, allowing us to control for some site‐specific anthropogenic factors that may affect acoustic abundance. Our data suggest that the progression of white‐nose syndrome is significantly affecting the acoustic abundance of bat species in the western Great Lakes region. Two species most susceptible to white‐nose syndrome have experienced significant declines within the 5 years of this study. Non‐susceptible species may also be impacted by white‐nose syndrome; declines in acoustic abundance for susceptible species may result in reduced competition or a foraging niche release, indirectly benefiting species less susceptible to white‐nose syndrome. Our results suggest that pup production or recruitment was not affected by white‐nose syndrome and that local summer habitat type may not be a factor influencing bat population resistance and resiliency to white‐nose syndrome in our study area. Our results also suggest that white‐nose syndrome is having a greater effect on bat populations at higher latitudes. Future studies should focus on the direct effects of white‐nose syndrome and its impact on various bat species populations. In addition to small scale studies, we also encourage the maintenance of long‐term monitoring of bat populations. Long‐term monitoring over large spatial scales can provide important insights for conservation decision‐makers.

## AUTHOR CONTRIBUTIONS

**Elyse C. Mallinger:** Conceptualization (equal); formal analysis (equal); methodology (equal); visualization (equal); writing – original draft (lead); writing – review and editing (equal). **Katy R. Goodwin:** Conceptualization (equal); data curation (lead); formal analysis (equal); methodology (equal); visualization (equal); writing – review and editing (equal). **Alan Kirschbaum:** Conceptualization (equal); data curation (equal); methodology (equal); visualization (equal); writing – review and editing (equal). **Yunyi Shen:** Conceptualization (equal); formal analysis (equal); visualization (equal); writing – review and editing (equal). **Erin H. Gillam:** Conceptualization (equal); writing – review and editing (equal). **Erik R. Olson:** Conceptualization (equal); formal analysis (equal); methodology (equal); supervision (equal); visualization (equal); writing – review and editing (equal).

## FUNDING INFORMATION

Funding for this research was provided by the National Park Service White‐Nose Syndrome project (GLNF CESU Agreement P16AC00340) and Northland College (Professional Development & Sabbatical Program; Department of Natural Resources; Sigurd Olson Professorship in the Natural Sciences; Raymond D Peters Professorship in biology).

## CONFLICT OF INTEREST STATEMENT

The authors declare there are no conflict of interest.

## Supporting information


Figure S1.

Appendix S1.

## Data Availability

All data are publicly available through the North American Bat Monitoring Program Database, v7.0.31, U.S. Geological Survey. Data may be accessed at https://www.nabatmonitoring.org/, by referencing GLKN Acoustic Monitoring as the project name and National Park Service as the contributing organization.
