# Supplementary material for: Species‐specific responses to white‐nose syndrome in the Great Lakes region
Source: Ecol Evol. 2023 Jul 9;13(7):e10267. doi: 10.1002/ece3.10267 (PMC10329912; doi:10.1002/ece3.10267)
Supplement: Supplementary file 1 — Figure S1. [file ECE3-13-e10267-s001.pdf]

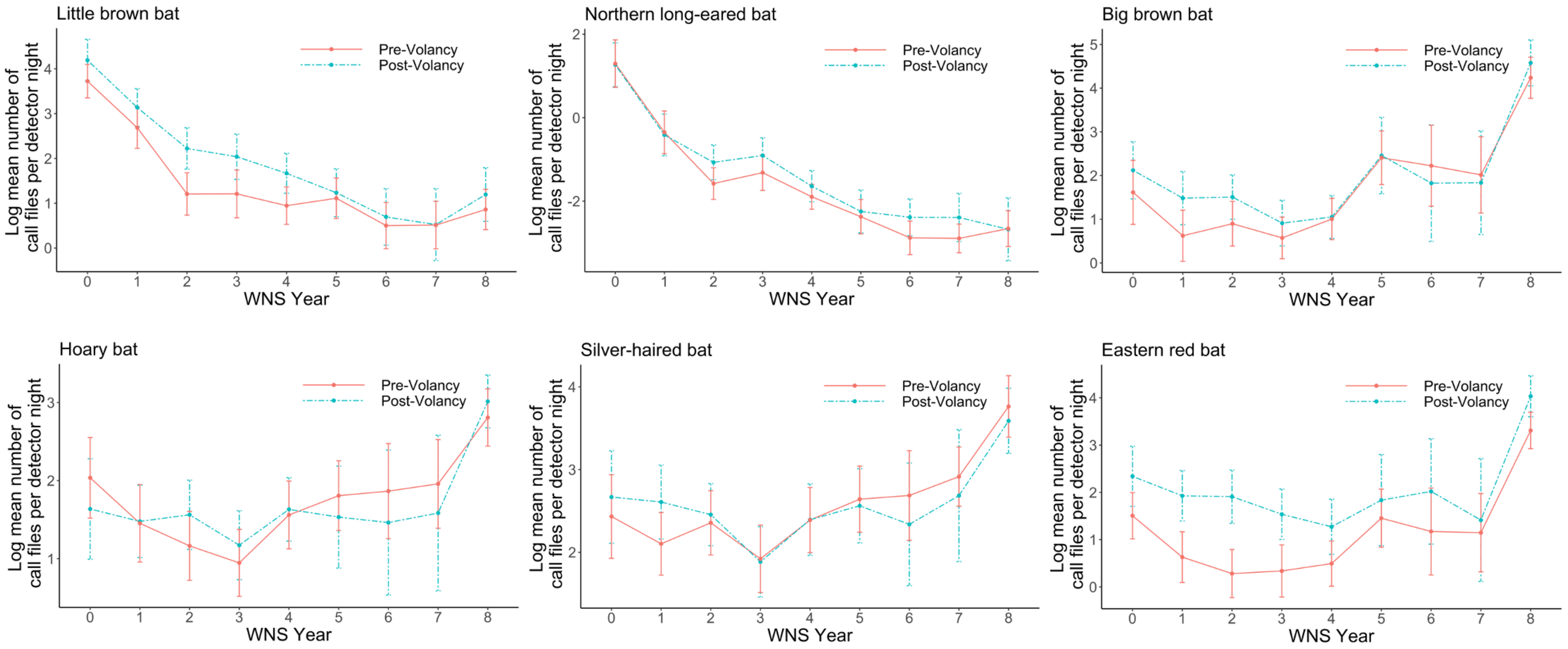
Little brown bat
Northern long-eared bat
Big brown bat
Hoary bat
Silver-haired bat
Eastern red bat
Pre-Volancy
Post-Volancy
Log mean number of call files per detector night
WNS Year
