# Supplementary material for: Species‐specific responses to white‐nose syndrome in the Great Lakes region
Source: Ecol Evol. 2023 Jul 9;13(7):e10267. doi: 10.1002/ece3.10267 (PMC10329912; doi:10.1002/ece3.10267)
Supplement: Supplementary file 2 — Appendix S1. [file ECE3-13-e10267-s002.docx]

**Appendix**

**Table S1.** List of *a priori* models used. * indicates interactive terms; + indicates additive terms. All models included a random effect for sample site location.

**Table S2.** Model averaged unconditional parameter estimates in order of importance for possible factors influencing the mean number of little brown bat (*Myotis lucifugus*) calls at a site per night across nine different national parks within the western Great Lakes region from 2016-2020.

| Variable | β coefficient | ± 95% confidence intervals | Lower CI | Upper CI |
| --- | --- | --- | --- | --- |
| Intercept | 0.000 | 0.000 | 0.000 | 0.000 |
| Pup (pre) | **-0.072** | **0.052** | **-0.123** | **-0.020** |
| WNS | **-0.318** | **0.169** | **-0.487** | **-0.149** |
| Park (GRPO)^a^ | **-0.176** | **0.131** | **-0.307** | **-0.045** |
| Park (INDU)^a^ | **-0.308** | **0.188** | **-0.496** | **-0.120** |
| Park (ISRO)^a^ | 0.046 | 0.165 | -0.119 | 0.211 |
| Park (MISS)^a^ | 0.015 | 0.128 | -0.113 | 0.143 |
| Park (PIRO)^a^ | **-0.452** | **0.152** | **-0.603** | **-0.300** |
| Park (SACN)^a^ | -0.107 | 0.125 | -0.232 | 0.018 |
| Park (SLBE) ^a^ | **-0.165** | **0.158** | **-0.322** | **-0.007** |
| Park (VOYA) ^a^ | -0.130 | 0.132 | -0.262 | 0.001 |
| Park (GRPO) ^a^ * WNS | **-0.133** | **0.079** | **-0.212** | **-0.055** |
| Park (INDU) ^a^ * WNS | 0.204 | 0.226 | -0.022 | 0.430 |
| Park (ISRO) ^a^ * WNS | **-0.259** | **0.179** | **-0.438** | **-0.080** |
| Park (MISS) ^a^ * WNS | -0.051 | 0.069 | -0.120 | 0.017 |
| Park (PIRO) ^a^ * WNS | -0.094 | 0.133 | -0.227 | 0.038 |
| Park (SACN) ^a^ * WNS | 0.043 | 0.079 | -0.036 | 0.121 |
| Park (SLBE) ^a^ * WNS | 0.067 | 0.138 | -0.071 | 0.205 |
| Park (VOYA) ^a^ * WNS | -0.015 | 0.080 | -0.095 | 0.064 |
| Habitat type (forest) | 0.025 | 0.149 | -0.123 | 0.174 |
| Habitat type (forest/open) | 0.016 | 0.098 | -0.082 | 0.113 |
| Habitat type (open) | 0.028 | 0.149 | -0.121 | 0.177 |
| Habitat type (woody wetland) | 0.020 | 0.113 | -0.093 | 0.133 |
| Park (GRPO) ^a^ * Pup (pre) | 0.000 | 0.000 | 0.000 | 0.000 |
| Park (INDU) ^a^ * Pup (pre) | 0.000 | 0.000 | 0.000 | 0.000 |
| Park (ISRO) ^a^ * Pup (pre) | 0.000 | 0.000 | 0.000 | 0.000 |
| Park (MISS) ^a^ * Pup (pre) | 0.000 | 0.000 | 0.000 | 0.000 |
| Park (PIRO) ^a^ * Pup (pre) | 0.000 | 0.000 | 0.000 | 0.000 |
| Park (SACN) ^a^ * Pup (pre) | 0.000 | 0.000 | 0.000 | 0.000 |
| Park (SLBE) ^a^ * Pup (pre) | 0.000 | 0.000 | 0.000 | 0.000 |
| Park (VOYA) ^a^ * Pup (pre) | 0.000 | 0.000 | 0.000 | 0.000 |
| Pup (pre) * WNS | 0.000 | 0.000 | 0.000 | 0.000 |
| Habitat type (forest) * WNS | 0.000 | 0.000 | 0.000 | 0.000 |
| Habitat type (forest/open) * WNS | 0.000 | 0.000 | 0.000 | 0.000 |
| Habitat type (open) * WNS | 0.000 | 0.000 | 0.000 | 0.000 |
| Habitat type (woody wetland) * WNS | 0.000 | 0.000 | 0.000 | 0.000 |
| Habitat type (forest) * Pup (pre) | 0.000 | 0.000 | 0.000 | 0.000 |
| Habitat type (forest/open) * Pup (pre) | 0.000 | 0.000 | 0.000 | 0.000 |
| Habitat type (open) * Pup (pre) | 0.000 | 0.000 | 0.000 | 0.000 |
| Habitat type (woody wetland) * Pup (pre) | 0.000 | 0.000 | 0.000 | 0.000 |

^a^GRPO= Grand Portage National Monument, INDU= Indiana Dunes National Park, ISRO= Isle Royale National Park, MISS= Mississippi National River and Recreation Area, PIRO= Pictured Rocks National Lakeshore, SACN= Saint Croix National Scenic Riverway, SLBE= Sleeping Bear Dunes National Lakeshore, VOYA= Voyageurs National Park.

**Table S3.** Model averaged unconditional parameter estimates in order of importance for possible factors influencing the mean number of northern long-eared bat (*Myotis septentrionalis*) calls at a site per night across nine different national parks within the western Great Lakes region from 2016-2020.

| Variable | β coefficient | ± 95% confidence intervals | Lower CI | Upper CI |
| --- | --- | --- | --- | --- |
| Intercept | 0.000 | 0.000 | 0.000 | 0.000 |
| Habitat type (forest) | 0.202 | 0.348 | -0.146 | 0.550 |
| Habitat type (forest/open) | 0.134 | 0.236 | -0.101 | 0.370 |
| Habitat type (open) | 0.116 | 0.216 | -0.100 | 0.332 |
| Habitat type (woody wetland) | 0.151 | 0.256 | -0.105 | 0.407 |
| Park (GRPO)^a^ | 0.038 | 0.135 | -0.097 | 0.173 |
| Park (INDU)^a^ | **-0.621** | **0.214** | **-0.835** | **-0.407** |
| Park (ISRO)^a^ | **0.367** | **0.183** | **0.184** | **0.550** |
| Park (MISS)^a^ | 0.012 | 0.144 | -0.131 | 0.156 |
| Park (PIRO)^a^ | **-0.317** | **0.173** | **-0.490** | **-0.145** |
| Park (SACN)^a^ | **-0.138** | **0.131** | **-0.269** | **-0.007** |
| Park (SLBE) ^a^ | **-0.386** | **0.175** | **-0.560** | **-0.211** |
| Park (VOYA) ^a^ | -0.106 | 0.135 | -0.240 | 0.029 |
| WNS | **-0.371** | **0.212** | **-0.583** | **-0.160** |
| Park (GRPO) ^a^ * WNS | **-0.254** | **0.099** | **-0.352** | **-0.155** |
| Park (INDU) ^a^ * WNS | **0.402** | **0.283** | **0.120** | **0.685** |
| Park (ISRO) ^a^ * WNS | **-0.453** | **0.224** | **-0.677** | **-0.229** |
| Park (MISS) ^a^ * WNS | -0.079 | 0.086 | -0.165 | 0.006 |
| Park (PIRO) ^a^ * WNS | -0.029 | 0.164 | -0.193 | 0.135 |
| Park (SACN) ^a^ * WNS | -0.037 | 0.098 | -0.135 | 0.061 |
| Park (SLBE) ^a^ * WNS | 0.204 | 0.173 | **0.031** | **0.376** |
| Park (VOYA) ^a^ * WNS | -0.005 | 0.099 | -0.104 | 0.094 |
| Pup (pre) | -0.004 | 0.028 | -0.032 | 0.023 |
| Pup (pre) * WNS | 0.000 | 0.000 | 0.000 | 0.000 |
| Habitat type (forest) * WNS | 0.000 | 0.001 | -0.001 | 0.001 |
| Habitat type (forest/open) * WNS | 0.000 | 0.000 | 0.000 | 0.000 |
| Habitat type (open) * WNS | 0.000 | 0.000 | 0.000 | 0.000 |
| Habitat type (woody wetland) * WNS | 0.000 | 0.001 | -0.001 | 0.001 |
| Park (GRPO) ^a^ * Pup (pre) | 0.000 | 0.000 | 0.000 | 0.000 |
| Park (INDU) ^a^ * Pup (pre) | 0.000 | 0.000 | 0.000 | 0.000 |
| Park (ISRO) ^a^ * Pup (pre) | 0.000 | 0.000 | 0.000 | 0.000 |
| Park (MISS) ^a^ * Pup (pre) | 0.000 | 0.000 | 0.000 | 0.000 |
| Park (PIRO) ^a^ * Pup (pre) | 0.000 | 0.000 | 0.000 | 0.000 |
| Park (SACN) ^a^ * Pup (pre) | 0.000 | 0.000 | 0.000 | 0.000 |
| Park (SLBE) ^a^ * Pup (pre) | 0.000 | 0.000 | 0.000 | 0.000 |
| Park (VOYA) ^a^ * Pup (pre) | 0.000 | 0.000 | 0.000 | 0.000 |
| Habitat type (forest) * Pup (pre) | 0.000 | 0.000 | 0.000 | 0.000 |
| Habitat type (forest/open) * Pup (pre) | 0.000 | 0.000 | 0.000 | 0.000 |
| Habitat type (open) * Pup (pre) | 0.000 | 0.000 | 0.000 | 0.000 |
| Habitat type (woody wetland) * Pup (pre) | 0.000 | 0.000 | 0.000 | 0.000 |

^a^GRPO= Grand Portage National Monument, INDU= Indiana Dunes National Park, ISRO= Isle Royale National Park, MISS= Mississippi National River and Recreation Area, PIRO= Pictured Rocks National Lakeshore, SACN= Saint Croix National Scenic Riverway, SLBE= Sleeping Bear Dunes National Lakeshore, VOYA= Voyageurs National Park.

**Table S4.** Model averaged unconditional parameter estimates in order of importance for possible factors influencing the mean number of big brown bat (*Eptesicus fuscus*) calls at a site per night across nine different national parks within the western Great Lakes region from 2016-2020.

| Variable | β coefficient | ± 95% confidence intervals | Lower CI | Upper CI |
| --- | --- | --- | --- | --- |
| Intercept | 0.000 | 0.000 | 0.000 | 0.000 |
| WNS | **0.102** | **0.039** | **0.063** | **0.140** |
| Park (GRPO)^a^ | -0.015 | 0.101 | -0.116 | 0.085 |
| Park (INDU)^a^ | **0.564** | **0.109** | **0.455** | **0.673** |
| Park (ISRO)^a^ | **-0.126** | **0.102** | **-0.228** | **-0.024** |
| Park (MISS)^a^ | **0.472** | **0.097** | **0.375** | **0.569** |
| Park (PIRO)^a^ | **-0.196** | **0.090** | **-0.286** | **-0.107** |
| Park (SACN)^a^ | **0.329** | **0.094** | **0.236** | **0.423** |
| Park (SLBE) ^a^ | **0.308** | **0.101** | **0.207** | **0.409** |
| Park (VOYA) ^a^ | 0.013 | 0.096 | -0.083 | 0.109 |
| Pup (pre) | **-0.099** | **0.099** | **-0.198** | **-0.001** |
| Park (GRPO) ^a^ * Pup (pre) | 0.027 | 0.072 | -0.045 | 0.099 |
| Park (INDU) ^a^ * Pup (pre) | -0.005 | 0.072 | -0.077 | 0.067 |
| Park (ISRO) ^a^ * Pup (pre) | 0.017 | 0.074 | -0.057 | 0.091 |
| Park (MISS) ^a^ * Pup (pre) | 0.051 | 0.074 | -0.022 | 0.125 |
| Park (PIRO) ^a^ * Pup (pre) | 0.046 | 0.073 | -0.027 | 0.119 |
| Park (SACN) ^a^ * Pup (pre) | 0.053 | 0.074 | -0.021 | 0.127 |
| Park (SLBE) ^a^ * Pup (pre) | 0.033 | 0.077 | -0.044 | 0.110 |
| Park (VOYA) ^a^ * Pup (pre) | -0.046 | 0.064 | -0.109 | 0.018 |
| Habitat type (forest) | -0.019 | 0.109 | -0.128 | 0.090 |
| Habitat type (forest/open) | -0.003 | 0.052 | -0.055 | 0.049 |
| Habitat type (open) | -0.001 | 0.053 | -0.053 | 0.052 |
| Habitat type (woody wetland) | -0.010 | 0.066 | -0.075 | 0.056 |
| Pup (pre) * WNS | 0.000 | 0.011 | -0.011 | 0.010 |
| Habitat type (forest) * Pup (pre) | 0.000 | 0.004 | -0.004 | 0.004 |
| Habitat type (forest/open) * Pup (pre) | 0.000 | 0.002 | -0.002 | 0.002 |
| Habitat type (open) * Pup (pre) | 0.000 | 0.003 | -0.003 | 0.003 |
| Habitat type (woody wetland) * Pup (pre) | 0.000 | 0.002 | -0.002 | 0.002 |
| Park (GRPO) ^a^ * WNS | 0.000 | 0.001 | -0.001 | 0.001 |
| Park (INDU) ^a^ * WNS | 0.000 | 0.006 | -0.006 | 0.006 |
| Park (ISRO) ^a^ * WNS | 0.000 | 0.003 | -0.003 | 0.003 |
| Park (MISS) ^a^ * WNS | 0.000 | 0.002 | -0.002 | 0.002 |
| Park (PIRO) ^a^ * WNS | 0.000 | 0.006 | -0.006 | 0.006 |
| Park (SACN) ^a^ * WNS | 0.000 | 0.001 | -0.001 | 0.001 |
| Park (SLBE) ^a^ * WNS | 0.000 | 0.003 | -0.003 | 0.003 |
| Park (VOYA) ^a^ * WNS | 0.000 | 0.001 | -0.001 | 0.001 |
| Habitat type (forest) * WNS | 0.000 | 0.000 | 0.000 | 0.000 |
| Habitat type (forest/open) * WNS | 0.000 | 0.000 | 0.000 | 0.000 |
| Habitat type (open) * WNS | 0.000 | 0.000 | 0.000 | 0.000 |
| Habitat type (woody wetland) * WNS | 0.000 | 0.000 | 0.000 | 0.000 |

^a^GRPO= Grand Portage National Monument, INDU= Indiana Dunes National Park, ISRO= Isle Royale National Park, MISS= Mississippi National River and Recreation Area, PIRO= Pictured Rocks National Lakeshore, SACN= Saint Croix National Scenic Riverway, SLBE= Sleeping Bear Dunes National Lakeshore, VOYA= Voyageurs National Park.

**Table S5.** Model averaged unconditional parameter estimates in order of importance for possible factors influencing the mean number of hoary bat (*Lasiurus cinereus*) calls at a site per night across nine different national parks within the western Great Lakes region from 2016-2020.

| Variable | β coefficient | ± 95% confidence intervals | Lower CI | Upper CI |
| --- | --- | --- | --- | --- |
| Intercept | 0.000 | 0.000 | 0.000 | 0.000 |
| Habitat type (forest) | -0.119 | 0.247 | -0.365 | 0.128 |
| Habitat type (forest/open) | -0.023 | 0.150 | -0.173 | 0.127 |
| Habitat type (open) | 0.029 | 0.159 | -0.130 | 0.188 |
| Habitat type (woody wetland) | -0.060 | 0.158 | -0.218 | 0.098 |
| WNS | **0.167** | **0.054** | **0.114** | **0.221** |
| Park (GRPO)^a^ | 0.053 | 0.120 | -0.067 | 0.173 |
| Park (INDU)^a^ | **0.277** | **0.140** | **0.137** | **0.417** |
| Park (ISRO)^a^ | **-0.162** | **0.124** | **-0.286** | **-0.039** |
| Park (MISS)^a^ | **0.265** | **0.124** | **0.141** | **0.389** |
| Park (PIRO)^a^ | **-0.179** | **0.097** | **-0.276** | **-0.082** |
| Park (SACN)^a^ | **0.373** | **0.111** | **0.262** | **0.484** |
| Park (SLBE) ^a^ | **0.193** | **0.123** | **0.070** | **0.315** |
| Park (VOYA) ^a^ | **0.125** | **0.118** | **0.007** | **0.243** |
| Pup (pre) | 0.002 | 0.031 | -0.029 | 0.033 |
| Pup (pre) * WNS | -0.002 | 0.026 | -0.028 | 0.024 |
| Park (GRPO) ^a^ * Pup (pre) | 0.000 | 0.010 | -0.010 | 0.010 |
| Park (INDU) ^a^ * Pup (pre) | 0.000 | 0.011 | -0.011 | 0.011 |
| Park (ISRO) ^a^ * Pup (pre) | 0.000 | 0.010 | -0.010 | 0.010 |
| Park (MISS) ^a^ * Pup (pre) | 0.001 | 0.018 | -0.017 | 0.018 |
| Park (PIRO) ^a^ * Pup (pre) | 0.000 | 0.010 | -0.009 | 0.010 |
| Park (SACN) ^a^ * Pup (pre) | 0.001 | 0.015 | -0.014 | 0.015 |
| Park (SLBE) ^a^ * Pup (pre) | 0.000 | 0.015 | -0.015 | 0.014 |
| Park (VOYA) ^a^ * Pup (pre) | 0.000 | 0.010 | -0.010 | 0.010 |
| Habitat type (forest) * Pup (pre) | 0.000 | 0.014 | -0.014 | 0.013 |
| Habitat type (forest/open) * Pup (pre) | 0.000 | 0.009 | -0.009 | 0.009 |
| Habitat type (open) * Pup (pre) | 0.000 | 0.009 | -0.009 | 0.009 |
| Habitat type (woody wetland) * Pup (pre) | 0.000 | 0.007 | -0.007 | 0.007 |
| Habitat type (forest) * WNS | 0.000 | 0.001 | -0.001 | 0.001 |
| Habitat type (forest/open) * WNS | 0.000 | 0.000 | 0.000 | 0.000 |
| Habitat type (open) * WNS | 0.000 | 0.000 | 0.000 | 0.000 |
| Habitat type (woody wetland) * WNS | 0.000 | 0.001 | -0.001 | 0.001 |
| Park (GRPO) ^a^ * WNS | 0.000 | 0.000 | 0.000 | 0.000 |
| Park (INDU) ^a^ * WNS | 0.000 | 0.000 | 0.000 | 0.000 |
| Park (ISRO) ^a^ * WNS | 0.000 | 0.000 | 0.000 | 0.000 |
| Park (MISS) ^a^ * WNS | 0.000 | 0.000 | 0.000 | 0.000 |
| Park (PIRO) ^a^ * WNS | 0.000 | 0.000 | 0.000 | 0.000 |
| Park (SACN) ^a^ * WNS | 0.000 | 0.000 | 0.000 | 0.000 |
| Park (SLBE) ^a^ * WNS | 0.000 | 0.000 | 0.000 | 0.000 |
| Park (VOYA) ^a^ * WNS | 0.000 | 0.000 | 0.000 | 0.000 |

^a^GRPO= Grand Portage National Monument, INDU= Indiana Dunes National Park, ISRO= Isle Royale National Park, MISS= Mississippi National River and Recreation Area, PIRO= Pictured Rocks National Lakeshore, SACN= Saint Croix National Scenic Riverway, SLBE= Sleeping Bear Dunes National Lakeshore, VOYA= Voyageurs National Park.

**Table S6.** Model averaged unconditional parameter estimates in order of importance for possible factors influencing the mean number of silver-haired bat (*Lasionycteris noctivagans*) calls at a site per night across nine different national parks within the western Great Lakes region from 2016-2020.

| Variable | β coefficient | ± 95% confidence intervals | Lower CI | Upper CI |
| --- | --- | --- | --- | --- |
| Intercept | 0.000 | 0.000 | 0.000 | 0.000 |
| Park (GRPO)^a^ | 0.073 | 0.140 | -0.067 | 0.212 |
| Park (INDU)^a^ | **0.350** | **0.197** | **0.153** | **0.547** |
| Park (ISRO)^a^ | **-0.329** | **0.171** | **-0.500** | **-0.158** |
| Park (MISS)^a^ | **0.321** | **0.141** | **0.180** | **0.462** |
| Park (PIRO)^a^ | 0.121 | 0.155 | -0.034 | 0.276 |
| Park (SACN)^a^ | **0.477** | **0.133** | **0.345** | **0.610** |
| Park (SLBE) ^a^ | **0.407** | **0.167** | **0.241** | **0.574** |
| Park (VOYA) ^a^ | 0.068 | 0.139 | -0.071 | 0.207 |
| WNS | **0.335** | **0.168** | **0.167** | **0.502** |
| Park (GRPO) ^a^ * WNS | 0.041 | 0.079 | -0.038 | 0.119 |
| Park (INDU) ^a^ * WNS | -0.208 | 0.225 | -0.432 | 0.017 |
| Park (ISRO) ^a^ * WNS | 0.093 | 0.178 | -0.085 | 0.271 |
| Park (MISS) ^a^ * WNS | -0.050 | 0.068 | -0.118 | 0.018 |
| Park (PIRO) ^a^ * WNS | **-0.353** | **0.132** | **-0.485** | **-0.221** |
| Park (SACN) ^a^ * WNS | -0.019 | 0.078 | -0.097 | 0.059 |
| Park (SLBE) ^a^ * WNS | **-0.271** | **0.137** | **-0.408** | **-0.134** |
| Park (VOYA) ^a^ * WNS | 0.039 | 0.079 | -0.040 | 0.117 |
| Habitat type (forest) | -0.049 | 0.193 | -0.242 | 0.144 |
| Habitat type (forest/open) | -0.020 | 0.121 | -0.141 | 0.100 |
| Habitat type (open) | 0.026 | 0.132 | -0.106 | 0.158 |
| Habitat type (woody wetland) | -0.014 | 0.112 | -0.125 | 0.098 |
| Pup (pre) | -0.001 | 0.015 | -0.016 | 0.014 |
| Park (GRPO) ^a^ * Pup (pre) | 0.000 | 0.000 | 0.000 | 0.000 |
| Park (INDU) ^a^ * Pup (pre) | 0.000 | 0.000 | 0.000 | 0.000 |
| Park (ISRO) ^a^ * Pup (pre) | 0.000 | 0.000 | 0.000 | 0.000 |
| Park (MISS) ^a^ * Pup (pre) | 0.000 | 0.000 | 0.000 | 0.000 |
| Park (PIRO) ^a^ * Pup (pre) | 0.000 | 0.000 | 0.000 | 0.000 |
| Park (SACN) ^a^ * Pup (pre) | 0.000 | 0.000 | 0.000 | 0.000 |
| Park (SLBE) ^a^ * Pup (pre) | 0.000 | 0.000 | 0.000 | 0.000 |
| Park (VOYA) ^a^ * Pup (pre) | 0.000 | 0.000 | 0.000 | 0.000 |
| Pup (pre) * WNS | 0.000 | 0.000 | 0.000 | 0.000 |
| Habitat type (forest) * WNS | 0.000 | 0.000 | 0.000 | 0.000 |
| Habitat type (forest/open) * WNS | 0.000 | 0.000 | 0.000 | 0.000 |
| Habitat type (open) * WNS | 0.000 | 0.000 | 0.000 | 0.000 |
| Habitat type (woody wetland) * WNS | 0.000 | 0.000 | 0.000 | 0.000 |
| Habitat type (forest) * Pup (pre) | 0.000 | 0.000 | 0.000 | 0.000 |
| Habitat type (forest/open) * Pup (pre) | 0.000 | 0.000 | 0.000 | 0.000 |
| Habitat type (open) * Pup (pre) | 0.000 | 0.000 | 0.000 | 0.000 |
| Habitat type (woody wetland) * Pup (pre) | 0.000 | 0.000 | 0.000 | 0.000 |

^a^GRPO= Grand Portage National Monument, INDU= Indiana Dunes National Park, ISRO= Isle Royale National Park, MISS= Mississippi National River and Recreation Area, PIRO= Pictured Rocks National Lakeshore, SACN= Saint Croix National Scenic Riverway, SLBE= Sleeping Bear Dunes National Lakeshore, VOYA= Voyageurs National Park.

**Table S7.** Model averaged unconditional parameter estimates in order of importance for possible factors influencing the mean number of eastern red bat (*Lasiurus borealis*) calls at a site per night across nine different national parks within the western Great Lakes region from 2016-2020.

| Variable | β coefficient | ± 95% confidence intervals | Lower CI | Upper CI |
| --- | --- | --- | --- | --- |
| Intercept | 0.000 | 0.000 | 0.000 | 0.000 |
| WNS | **-0.176** | **0.048** | **-0.224** | **-0.128** |
| Park (GRPO)^a^ | **-0.261** | **0.116** | **-0.376** | **-0.145** |
| Park (INDU)^a^ | **0.376** | **0.127** | **0.249** | **0.503** |
| Park (ISRO)^a^ | **-0.288** | **0.119** | **-0.407** | **-0.169** |
| Park (MISS)^a^ | 0.055 | 0.109 | -0.054 | 0.164 |
| Park (PIRO)^a^ | **-0.375** | **0.100** | **-0.475** | **-0.276** |
| Park (SACN)^a^ | 0.009 | 0.104 | -0.095 | 0.113 |
| Park (SLBE) ^a^ | **0.171** | **0.118** | **0.053** | **0.288** |
| Park (VOYA) ^a^ | **-0.137** | **0.107** | **-0.244** | **-0.030** |
| Pup (pre) | **-0.199** | **0.120** | **-0.318** | **-0.079** |
| Park (GRPO) ^a^ * Pup (pre) | 0.007 | 0.087 | -0.080 | 0.094 |
| Park (INDU) ^a^ * Pup (pre) | 0.081 | 0.092 | -0.010 | 0.173 |
| Park (ISRO) ^a^ * Pup (pre) | **0.162** | **0.093** | **0.069** | **0.254** |
| Park (MISS) ^a^ * Pup (pre) | 0.066 | 0.078 | -0.012 | 0.144 |
| Park (PIRO) ^a^ * Pup (pre) | 0.037 | 0.081 | -0.044 | 0.118 |
| Park (SACN) ^a^ * Pup (pre) | **0.121** | **0.078** | **0.044** | **0.199** |
| Park (SLBE) ^a^ * Pup (pre) | -0.010 | 0.092 | -0.102 | 0.082 |
| Park (VOYA) ^a^ * Pup (pre) | -0.035 | 0.067 | -0.102 | 0.032 |
| Habitat type (forest) | -0.026 | 0.132 | -0.157 | 0.106 |
| Habitat type (forest/open) | -0.019 | 0.097 | -0.116 | 0.078 |
| Habitat type (open) | 0.001 | 0.072 | -0.070 | 0.073 |
| Habitat type (woody wetland) | -0.020 | 0.097 | -0.117 | 0.077 |
| Park (GRPO) ^a^ * WNS | 0.000 | 0.009 | -0.009 | 0.009 |
| Park (INDU) ^a^ * WNS | 0.000 | 0.011 | -0.011 | 0.011 |
| Park (ISRO) ^a^ * WNS | 0.000 | 0.020 | -0.020 | 0.019 |
| Park (MISS) ^a^ * WNS | 0.000 | 0.003 | -0.003 | 0.003 |
| Park (PIRO) ^a^ * WNS | 0.000 | 0.011 | -0.011 | 0.010 |
| Park (SACN) ^a^ * WNS | 0.000 | 0.002 | -0.002 | 0.002 |
| Park (SLBE) ^a^ * WNS | 0.000 | 0.013 | -0.013 | 0.012 |
| Park (VOYA) ^a^ * WNS | 0.000 | 0.005 | -0.005 | 0.005 |
| Pup (pre) * WNS | 0.000 | 0.004 | -0.004 | 0.004 |
| Habitat type (forest) * WNS | 0.000 | 0.003 | -0.003 | 0.003 |
| Habitat type (forest/open) * WNS | 0.000 | 0.003 | -0.003 | 0.003 |
| Habitat type (open) * WNS | 0.000 | 0.002 | -0.002 | 0.002 |
| Habitat type (woody wetland) * WNS | 0.000 | 0.003 | -0.003 | 0.003 |
| Habitat type (forest) * Pup (pre) | 0.000 | 0.004 | -0.004 | 0.004 |
| Habitat type (forest/open) * Pup (pre) | 0.000 | 0.003 | -0.003 | 0.003 |
| Habitat type (open) * Pup (pre) | 0.000 | 0.003 | -0.003 | 0.003 |
| Habitat type (woody wetland) * Pup (pre) | 0.000 | 0.003 | -0.003 | 0.003 |

^a^GRPO= Grand Portage National Monument, INDU= Indiana Dunes National Park, ISRO= Isle Royale National Park, MISS= Mississippi National River and Recreation Area, PIRO= Pictured Rocks National Lakeshore, SACN= Saint Croix National Scenic Riverway, SLBE= Sleeping Bear Dunes National Lakeshore, VOYA= Voyageurs National Park.
